# Pericytes: jack-of-all-trades in cancer-related inflammation

**DOI:** 10.3389/fphar.2024.1426033

**Published:** 2024-07-17

**Authors:** Marianna Moro, Federica Carolina Balestrero, Ambra A. Grolla

**Affiliations:** Department of Pharmaceutical Sciences, Università del Piemonte Orientale, Novara, Italy

**Keywords:** pericytes, cancer-related inflammation, tumor microenvironment, metastasis, immune evasion

## Abstract

Pericytes, recognized as mural cells, have long been described as components involved in blood vessel formation, playing a mere supporting role for endothelial cells (ECs). Emerging evidence strongly suggests their multifaceted roles in tissues and organs. Indeed, pericytes exhibit a remarkable ability to anticipate endothelial cell behavior and adapt their functions based on the specific cells they interact with. Pericytes can be activated by pro-inflammatory stimuli and crosstalk with immune cells, actively participating in their transmigration into blood vessels. Moreover, they can influence the immune response, often sustaining an immunosuppressive phenotype in most of the cancer types studied. In this review, we concentrate on the intricate crosstalk between pericytes and immune cells in cancer, highlighting the primary evidence regarding pericyte involvement in primary tumor mass dynamics, their contributions to tumor reprogramming for invasion and migration of malignant cells, and their role in the formation of pre-metastatic niches. Finally, we explored recent and emerging pharmacological approaches aimed at vascular normalization, including novel strategies to enhance the efficacy of immunotherapy through combined use with anti-angiogenic drugs.

## 1 Introduction

Eberth in 1871 and Rouget in 1873 first described a subpopulation of cells as a cluster of contractile cells that extend their projections encircling the capillaries (Eberth, 1871; Rouget, 1874). Some years later, Zimmerman named them “pericytes,” on the basis of their localization along the vasculature, bypassing their many features too complicated to include in a unique name.

It is well established that these are multitasking cells, able to acquire different roles depending on the tissue or organ in which they are found and the cells they collaborate with.

In the last few years, the need for knowledge on pericytes has increased exactly as, several years ago, astrocytes started to be considered active cells in the brain and not simply structural tools for neurons. Indeed, recent data have described pericytes as essential orchestrators that anticipate endothelial cell (EC) behavior, and emerging evidence reveals novel pathological roles beyond the implications in angiogenesis.

This review aims to recapitulate the several activities of this heterogeneous population of contractile cells called pericytes, beyond their main role as mural cells that support endothelial vessel stability, and focus on their immune-modulatory activity, specifically in cancer. Last but not the least, we aim to highlight the still unmet knowledge regarding these multifaceted cells and their crosstalk with immune cells, believing in the potential of a pericyte-based therapy to improve current cancer immunotherapy.

## 2 Established knowledge about pericyte physiology in vessels

Pericytes might be defined as a jack-of-all-trade cell type, able to coordinate at least four main biological processes: i) central nervous system (CNS) homeostasis and blood–brain barrier (BBB) regulation; ii) angiogenesis and blood vessel stabilization; iii) tissue repair and regeneration; and iv) blood flow regulation. Emerging evidence shows how pericytes may recruit immune cells and play an active role in their transmigration into vessels, thus stating that a fifth function may be the regulation of immune responses in several immune-pathological contexts, as discussed below.

Pericytes are ubiquitously lining the endothelial cells in all microvessels across the organism, mainly abundant in capillaries. They send out primary cytoplasmic extensions along the outer surface of the endothelial tube. These extensions typically stretch across multiple ECs and sometimes connect adjacent capillary branches. Pericytes and ECs communicate by direct physical contact and paracrine/autocrine signaling pathways. Specifically, gap junctions containing cell-adhesion molecules such as N-cadherin, β-catenin, or extracellular matrix molecules such as fibronectin provide direct connections between the cytoplasm of pericytes and ECs (Cuevas et al., 1984).

As mentioned before, pericytes are crucial in the construction of the vascular wall, upkeep of the vascular barrier, assurance of blood vessel stability, and contribution to the maintenance of overall homeostasis in the human body (Holm et al., 2018).

However, the scenario is complicated by the multitasking abilities of pericytes to exert tissue-/organ-specific functions by heterogeneously expressing several molecules. To overcome the tissue-specific demands, pericyte abundance and distribution vary among organs. The microvasculature of the CNS has a higher EC–pericyte ratio (1:4 to 1:1) due to the presence of the BBB, of which they are one of the main components.

This is the reason why the physiology of these cells is mostly described in tissues belonging to the CNS, which might not resemble those of other organs in which the density of pericytes is reduced (Baek et al., 2022).

Within the CNS, pericytes are situated within the basement membrane, positioned between endothelial cells and astroglial endfeet. Regardless of their morphological diversity, pericyte coverage of the CNS microvasculature is approximately 80% in regions such as the cerebral cortex, hippocampus, striatum, midbrain, and cerebellum (Bell et al., 2010; Villaseñor et al., 2017), while it stands at approximately 50% in the spinal cord (Winkler et al., 2012). In peripheral regions, the pericyte-to-endothelial cell ratio ranges from 1:10 in the skin and lungs to 1:100 in the human skeletal muscle (Díaz-Flores et al., 2009).

Contrary to what was believed a few years ago, pericyte growth precedes the expansion of ECs: by rapidly proliferating, pericytes ensure the production of EC growth factors (Figueiredo et al., 2020). Pericytes coordinate and anticipate EC behavior through the secretion of a variety of growth factors, including cytokines and chemokines.

Under basal conditions, they release the vascular endothelial growth factor (VEGF) to control EC proliferation and microtubule formation through the activation of the VEGF receptor (VEGFR) expressed on ECs (Eilken et al., 2017). Moreover, angiopoietin-1 (Ang-1) is a paracrine ligand expressed by pericytes and a strong TIE2 agonist that supports endothelial cell survival, vessel stability, and endothelial barrier function (Saharinen et al., 2017). Tie2 is a receptor tyrosine kinase heavily enriched in the vascular endothelium whose tonic signaling actively maintains vascular quiescence. On the same axis, angiopoietin 2 (Ang-2) coordinates vessel disruption and EC death (Miners et al., 2023), while its role still remains controversial (Thurston et al., 2012).

Molecules involved in extracellular matrix remodeling, such as VCAM-1, ICAM-1, MMP2, and MMP9, are released by pericytes to coordinate vessel maturation and expansion, guiding EC migration (Su et al., 2019). Moreover, under basal conditions, they express several neurotrophic factors, such as nerve growth factor (NGF), brain-derived neurotrophic factor (BDNF), glial cell-derived neurotrophic factor (GDNF), and hepatocyte growth factor (HGF) (Shimizu et al., 2011). On the other hand, platelet-derived growth factor (PDGF)-BB is released by ECs to recruit pericytes via PDGF receptor-β (PDGFRβ) signaling to sustain their adhesion and spatial disposition in vasculogenic tube assembly and stabilization. Blockade of pericyte recruitment causes a lack of basement membrane matrix deposition and, concomitantly, increased vessel widths (Stratman et al., 2010).

In pericyte physiology, fibroblast-growth factor (FGF) plays a crucial role in regulating their main signaling, including the PDGFR pathway. Specifically, FGF-2 is a potent pericyte-stimulating factor; by binding to FGFR2, it induces pericyte proliferation and orchestrates PDGFRβ signaling for vascular recruitment, as demonstrated in the tumor context (Hosaka et al., 2018).

Pericytes respond to different stimuli by changing their basal secretome. Under inflammatory conditions, such as under LPS, TNFα, or IL-1β stimuli or under tumor microenvironment (TME) influence, pericytes start to release inflammatory cytokines to recruit immune cells (IL-6, IL-8, CXCL1, CXCL2, CXCL3, CX3CL1, CCL5, and CCL2) and upregulate the adhesion molecules ICAM-1 and VCAM-1 (Gaceb et al., 2018). The Ang–Tie axis plays an essential role in inflammation. Ang2–Tie2 signaling induces the expression of ICAM-1 and VCAM-1, promoting leukocyte adhesion and migration to inflamed tissues in response to inflammatory cytokines (Fiedler et al., 2006). The involvement of pericyte-derived mediators in inflammation is further elucidated in the latter part of the review.

## 3 Complexities in pericyte markers

A major obstacle to studying pericytes lies in the absence of a universally accepted definition to differentiate them from other types of mural cells. Pericytes are identified by i) their location around vessels and their unique morphology, characterized by an oval-shaped cell body and elongated processes that encircle vascular structures; ii) concomitant expression of the accredited markers found highly expressed in the majority of all the described tissue-subtype pericytes. The combination of these features correctly identifies this type of cell.

In the mature brain, the usual indicators used to identify pericytes associated with capillaries include desmin, neural/glial antigen 2 (NG2), cluster of differentiation (CD) 13, CD146, and PDGFR-β (Nehls and Drenckhahn, 1991; Daneman et al., 2010; Smyth et al., 2018; Yamazaki and Mukouyama, 2018). Traditional cytoskeletal markers utilized for pericyte identification encompass non-muscle myosin (Joyce et al., 1985), nestin, and vimentin (Bandopadhyay et al., 2001).

Single-cell genome-wide quantitative transcriptomic analyses have revealed the molecular profile of brain pericytes (He et al., 2018; Vanlandewijck et al., 2018; Zeisel et al., 2018; Hariharan et al., 2020). Brain pericytes notably express various members of the solute carrier family (SLC), the ATP-binding cassette family, and ATP transporters, distinguishing them from lung pericytes. This highlights the organ-specific specialization of brain pericytes; indeed, depending on their lineage, pericytes may express lineage-related markers. For instance, mesoderm-derived pericytes retain CD45 and CD11b hematopoietic markers (Ozerdem, 2006), while pericytes in the brain, thymus, lungs, heart, liver, and gastrointestinal tract that originate from the ectoderm do not express these proteins.

To augment the complexity, some of the described markers are also expressed by other cell types; for example, PDGFRβ is also highly expressed by fibroblasts and smooth muscle cells (SMCs), or NG2 is even expressed by progenitors and mature oligodendrocytes in the CNS, as reviewed in Bhattacharya et al. (2020). This is the reason why only the concomitant expression of more markers and their localization next to the ECs may ascertain whether they are pericytes.

In adult neuropathological occurrences, pericytes demonstrate a transient retention of GFAP expression (Sharma et al., 2012). In mouse embryos, the presence of vimentin and regulator of G-protein signaling 5 (RGS5) expression indicates the presence of pericytes encircling developing arteries, with desmin and α-SMA remaining undetectable during this period. Nevertheless, RGS5 expression decreases after birth (Bandopadhyay et al., 2001; Bondjers et al., 2003).

Recent advancements in single-cell RNA sequencing have revealed significant differences in gene expression among pericytes from different organs, indicating distinct functional roles. In this regard, Mariona Graupera’s group conducted a comprehensive review of the current situation on pericyte markers, utilizing information provided by single-cell RNA sequencing data (van Splunder et al., 2024).

Seung-Han Baek and co-authors conducted an analysis of single-cell RNA sequencing data from tissue-specific mouse pericyte populations established by the Tabula Muris Senis to detect pericyte-specific markers. Within the murine lung, heart, kidney, and bladder, they identified a cluster of mural cells expressing either CSPG4 or PDGFR-β and established known pericyte markers. Differentially expressed genes from CSPG4/PDGFR-β expressing cells compared to non-expressing cells revealed 18 lung-specific, 4 heart-specific, 22 kidney-specific, and 4 bladder-specific pericyte markers. Comparative single-cell differential expression gene analysis highlighted potential pericyte marker candidates, such as Kcnk3 (lung), Rgs4 (heart), Myh11 and Kcna5 (kidney), Pcp4l1 (bladder), and Higd1b (lung and heart). Notably, validation of these markers was performed using the Human Lung Cell Atlas and human heart single-cell RNAseq databases, revealing the conservation of markers between mouse and human heart and lung pericytes (Baek et al., 2022).

Moreover, the analysis of single-cell RNA expression profiles derived from different human and mouse brain regions using a high-throughput and low-cost single-cell transcriptome sequencing method called EasySci revealed that highly pericyte-enriched expression was notable for SLC6A12 and SLC19A1. The immunohistochemical technique of staining for both antibodies was strongly positive in small blood vessels and was far more effective than a PDGFR-β antibody at staining pericyte-like cells in human brain sections (Gurler et al., 2023; Sziraki et al., 2023).

Exploring whether this diversity extends to pericytes in other tissues remains largely undiscovered. The identification of distinct tissue-specific markers for pericytes could greatly enrich our comprehension of their diversity and shed light on their significance in both health and disease contexts.

## 4 Pericytes have multipotent cell-like properties

The developmental origin of pericytes is heterogeneous, and much remains to be deciphered. Most commonly described is their origin from mesenchymal stem cells (MSCs). Genetic lineage tracing experiments using neural crest-specific Cre recombinase lines such as Wnt-1-Cre and Sox10-Cre mice in combination with a Cre-mediated reporter line demonstrate that the neural crest contributes to pericytes in the face, brain, and thymus (Korn et al., 2002; Foster et al., 2008; Müller et al., 2008). Using similar genetic lineage tracing experiments, the origin of pericytes in the gut (Wilm et al., 2005), lung (Que et al., 2008), and liver (Asahina et al., 2011) in mice has been traced to the mesothelium, a single layer of squamous epithelium. In the heart, the epicardial mesothelium gives rise to coronary pericytes and vascular smooth muscle cells (Zhou et al., 2008).

These studies clearly indicate that the origin of pericytes is heterogeneous in a tissue- and context-dependent manner.

Pericytes typically maintain a state of quiescence within an established microvessel network. Nonetheless, under various physiological stimuli, such as capillary network expansion or in response to pathological conditions, they undergo activation and proliferation. This response can lead to either self-renewal or differentiation. Our understanding of the molecular pathways governing the transition of pericytes between quiescence, proliferation, or differentiation remains limited.

Past literature sustains the role of the transforming growth factor beta (TGFβ) signaling pathway in the induction of undifferentiated progenitor cells into PDGFRβ-positive pericyte precursors, which are then attracted by PDGFβ-expressing endothelial cells (Creazzo et al., 1998; Hellstrom et al., 1999). TGFβ regulates the proliferation and differentiation of both mural cells and endothelial cells; however, its activation requires interaction between the two cell types (Sato and Rifkin, 1989).

The preservation of stemness and multipotent capabilities of pericytes relies heavily on their interactions with the basement membrane protein, specifically laminin, without which brain pericytes adopt the characteristics of contractile cells (Yao et al., 2014). Additionally, the maintenance of pericyte stemness is influenced by various other microenvironmental signals and transcription factors known to regulate the genes encoding the components of metabolic pathways.

When cultured *in vitro*, and this is an important aspect to highlight, pericytes demonstrate the ability to differentiate into multiple lineages, including osteoblasts, chondrocytes, and adipocytes (Crisan et al., 2008; Supakul et al., 2019). The presence of the transcription factor Runx2 (Osf2/Cbf1a) serves as a distinguishing feature of pericytes primed for osteogenic or chondrogenic differentiation (Farrington-Rock et al., 2004). In regard to pericyte-derived adipocytes, the expression of PPARγ, a transcription factor which is a pivotal regulator of pre-adipocyte differentiation, is induced when cultured pericytes are exposed to adipogenic factors (Lefterova et al., 2014).

Pericytes have the ability to also differentiate into vascular smooth muscle cells, myofibroblasts, and various parenchymal cells such as skeletal and cardiac myocytes. TGFβ/Notch signaling augment pericyte differentiation into smooth muscle cells (Volz et al., 2015) and into myofibroblasts (Kirton et al., 2006; Cheng et al., 2008; Aimaiti et al., 2019). Nakagomi’s group suggested that vascular pericytes acquire multipotent vascular stem cell activity under pathological conditions and may thus be a novel source of microglia (Sakuma et al., 2016). An interesting work demonstrated that pericyte hallmark-expressing cells from the adult human cerebral cortex can be transformed into neuronal cells through retrovirus-mediated co-expression of the transcription factors Sox2 and Mash1. These reprogrammed neuronal cells demonstrate the capacity for repetitive action potential firing and act as synaptic targets for other neurons, illustrating their ability to integrate into neural networks (Karow et al., 2012).

This broad multipotency has led researchers to suggest that pericytes may be the primary source of tissue-resident mesenchymal progenitors. Furthermore, their multipotent characteristics have fueled their increasing use in cell therapies aimed at promoting tissue healing and regeneration. In this regard, a very recent work underlines the importance of MSCs in regenerative medicine research and highlights the limitations in the number of MSCs available in the human body. Therefore, they explore pericytes as alternative regenerative cell sources as a substitute for human bone marrow-derived mesenchymal stem cells (hBM-MSCs) (Polat et al., 2024).

Nonetheless, it is important to underline the ability of pericytes to undergo pericyte-to-fibroblast transition (PFT), which contributes to inflammatory-associated fibrosis. The crosstalk between microvascular endothelial cells and pericytes promotes PFT through the PDGF-BB/PDGFRβ signaling pathway, which, if inhibited by imatinib, rescues fibrotic scarring and reduces the inflammation in spinal cord injury (Yao et al., 2022).

In this regard, TGF-β promotes pericyte–fibroblast transition in subretinal fibrosis through the Smad2/3 and Akt/mTOR pathways (Zhao et al., 2022).

However, multipotency is not a universal trait among all pericytes. Indeed, pericytes expressing the T-Box transcription factor 18 (Tbx18) show a lack of ability for trans-differentiation *in vivo* across multiple tissues examined, including skeletal, cardiac, and adipose tissues (Guimarães-Camboa et al., 2017). These findings challenge the prevailing perspective regarding endogenous pericytes as multipotent tissue-resident progenitors, suggesting that the observed plasticity, whether *in vitro* or following transplantation *in vivo*, might represent an artifact from cell manipulations conducted *ex vivo*. Therefore, other efforts have to be made to define pericytes as multipotent cells in a complex system such as the entire organism.

## 5 Immunomodulatory role of pericytes in cancer

Tumor angiogenesis is one of the main hallmarks of cancer, which provides the oxygen and nutrition supplies required for cancer progression. Even in cancer, ECs release PDGF-B that induces pericyte recruitment in the growing tumor vasculature by activating PDGFRβ signaling. On the other hand, endothelial-derived nitric oxide (NO) stimulates the production of VEGF-A in pericytes, which sustains EC proliferation and survival (Nishida et al., 2006; Meng et al., 2015). Normal pericyte–EC interaction is essential in maintaining vascular stability and normal microcirculation, but in cancer, TME influence completely overturns the phenotype, morphology, and localization of these cells, leading to abnormal, leaky, and unstructured vessels that fail to sustain nutrient and oxygen demand. In the current literature, the debate regarding whether a tumor with lower or higher pericyte coverage is more aggressive remains unresolved. Certainly, the change in the EC–pericyte ratio leads to dysregulated angiogenesis, typical of the tumor.

As mentioned before, pericytes release different pro-inflammatory chemokines and cytokines in response to pro-inflammatory stimuli (Navarro et al., 2016).

In the complex landscape of the TME, pericytes mutually interact through several signaling pathways with the different components of the TME (as outlined in Table 1), participating in i) EC proliferation; ii) tumor vascularization; iii) immune tolerance; iv) phenotypic transition; v) extravasation process; and vi) pre-metastatic niche formation.

**TABLE 1:** Signaling pathways regulate pericyte interaction with immune cells.

Signalling	Stimuli/context	Immune cell interaction	Biological effect	Reference
PDGFR-β	PDGF-BB tumors	TAM recruitment via IL-33 secretion by pericytes	Increase in metastasis via MMP9 produced by TAM	Yang et al., 2016; Andersson et al., 2018
	Glioma-derived pericytes	CECR-1 expression by macrophages	Pro-angiogenic activity via ECM component modulation	Zhu et al. (2017)
PDGFR-α	Mammary carcinoma-derived pericytes	PDGF-CC Lyve-1 TAM	Tumor growth	Opzoomer et al. (2021)
FGFR-1	FGF-2 from tumors	TAM recruitment via CXCL14-secretion	Increase in metastasis	Wang et al. (2022)
MCAM	Human microvascular pericytes	M2 macrophage polarization	MCAM/C1D63 prognostic signature in GBM	Zhang et al. (2022)
sGC	Lewis lung carcinoma-derived pericytes	sGC deletion promotes M2 macrophage polarization mediated by MIF	Promotion of immune evasion	Zhu et al. (2024)
CD80, CD86, and MHC-II	IL-6 from the TME	CD4^+^ T cell anergy with a decreased secretion of IL-4 and IFN-Υ	Pericytes as immunosuppressive weapons	Bose et al. (2013)
RGS-5	Melanoma tumour-derived pericytes	CD4^+^ T cell anergy mediated by tumor-derived pericyte-ICAM1 upregulation	Pericytes as immunosuppressive weapons	Bose et al. (2013)
	TGF-β from the TME	CD4^+^ T cell anergy	Irregular vascularization and NLPG treatment restore T-cell functionality, leading to RGS-5^high^ pericyte apoptosis	Dasgupta et al. (2022)
Endosialin	RCC-derived pericytes	Reduction in CD8^+^ T-cell infiltration	Anti-EN improves immune-checkpoint blockade therapy in RCC	Lu et al. (2023)
MFG-E8	NG2^+^ melanoma-derived MSCs	M2 macrophage activation	Tumor growth	Yamada et al. (2016)
IL-10	Glioma-derived pericytes	CD4^+^ T-cell inhibition with a decreased secretion of IL-2	Tumor growth	Valdor et al. (2017)
	Glioma-derived pericytes upregulating chaperone-mediated autophagy (CMA)	CD4^+^ T-cell inactivation decreasing IL-2 production	CMA-pericyte ablation reduces tumor growth	Valdor et al., 2019; Molina et al., 2022
TGF-β	Glioma-derived pericytes	CD4^+^ T-cell inhibition with a decreased secretion of IL-2	Tumor growth	Valdor et al. (2017)
	Glioma-derived pericytes	T-cell inhibition	Immunosuppressive pericytes	Ochs et al. (2013)
	Glioma-derived pericyte upregulating CMA	CD4^+^ T-cell inactivation decreasing IL-2 production	CMA-pericyte ablation reduces tumor growth	Valdor et al., 2019; Molina et al., 2022
CXCL9	Primary central nervous system lymphoma-derived pericytes	CD8^+^ T-cell and B-cell recruitment following CXCL9-CXCL12 heterocomplex formation	The perivascular microenvironment regulates immune effector cells	Venetz et al. (2010)
Pericyte coverage	MMP-9 from neutrophils	Tumor-associated neutrophils	Enlarged vessel partially covered by pericytes	Deryugina et al. (2014)
	IL-6 in TME	Decrease in MDSCs	Favorable outcome	Hong et al. (2015)
	MMP-9 from MDSCs in pre-metastatic niche	Gr-1^+^CD11b^+^ MDSCs	Aberrant metastatic vasculature formation	Yan et al. (2010)

**FIGURE 1:**
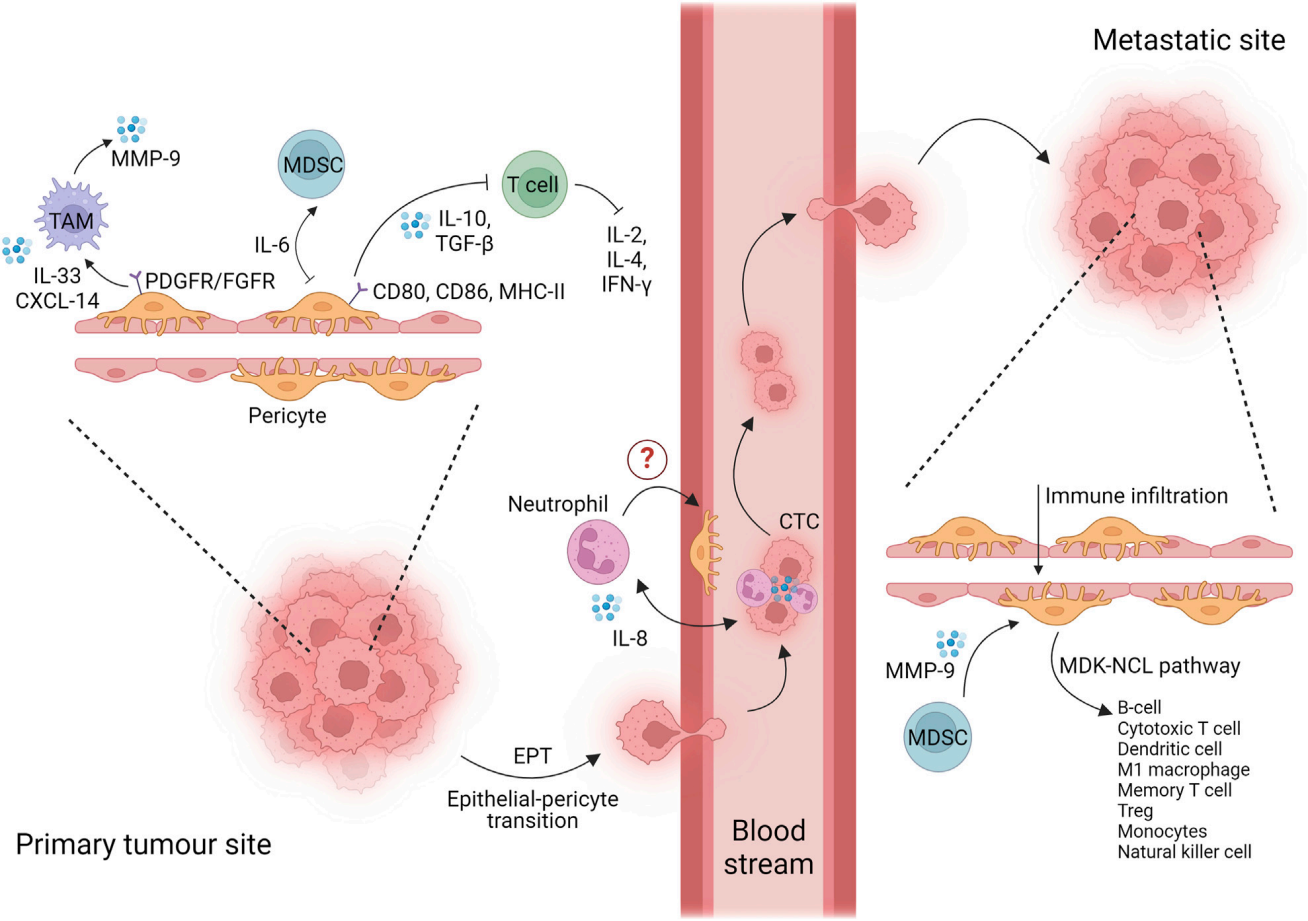
Interaction of pericytes and immune cells at every stage of tumor progression. Created with BioRender.com.

In recent years, the immunomodulatory role of tumor-derived pericytes has gained attention in relation to inflammation-associated tumor progression and invasion. Considering the pericyte plasticity among the different tissues and tumor types, it is not surprising to observe the intertwined role of pro-inflammatory pericytes communicating with various cell components of the TME. The main findings in this regard are reviewed in the following sections, compartmentalized based on the tumor progression steps: i) primary mass, ii) phenotype transition, and iii) metastatic niche, as shown in Figure 1.

### 5.1 Pericytes and cancer-related inflammation in the primary mass

#### 5.1.1 EC–pericyte interaction

Tumor branching is chaotic in the TME, and endothelial cell–pericyte interactions are characterized by altered signaling that provokes tumor progression and invasion (Sweeney et al., 2016; Sun et al., 2021).

Among the molecules involved in vessel formation, the Ang–Tie2 pathway emerged as a critical regulator in tumor angiogenesis. In different tumor models (such as glioma, Lewis lung carcinoma, and spontaneous mammary carcinoma), treatment with an antibody that simultaneously activates Tie-2 and inhibits Ang-2 (ABTAA) was useful in restoring pericyte PDGFR-β coverage and tightening endothelial cell junctions by increasing VE-cadherin and claudin-5 expression, promoting the stabilization of pericyte–endothelial cell contact. Moreover, it has been explored that ABTAA favors immune cell infiltration by reducing M2 polarization, promoting M1 infiltration, and simultaneously reducing T regulatory cell recruitment, contributing to tumor growth and metastasis suppression (Park et al., 2017).

Interestingly, integrin modulation could affect tumor growth without interfering with blood vessel functionality. The selective knockout of β3 integrin in pericytes has been shown to promote tumor growth via focal adhesion molecule (FAK)-Akt-NF-kβ activation toward the secretion of paracrine factors such as CXCL1, TIMP-1, and CCL2, which mediate cancer proliferation toward the MEK1-ERK1/2-ROCK2 axis without interfering with blood vessel density, perfusion, or tumor hypoxia (Wong et al., 2020). In this regard, pericyte-FAK loss has been shown to promote blood vessel formation by decreasing pericyte association with ECs, enhancing tumor proliferation via the Gas6-Axl-Akt pathway (Lechertier et al., 2023).

Furthermore, metabolic reprogramming impacts the vascular cell phenotype in tumors. Combining proteomic and metabolic flux analyses, hexokinase-2 has been found to drive glycolysis in tumor-derived pericytes. Pericyte-hexokinase-2, via ROCK2 and MLC2, abnormally interacts with ECs, leading to abnormal tubular formation when co-cultured with ECs *in vitro*. The delivery of chemotherapeutic agents, such as doxorubicin and hexokinase inhibitors, generates tumor blood vessels with enlarged diameter and perfusion and increased collagen IV expression, enhancing drug delivery without interfering with pericyte coverage (Meng et al., 2021).

#### 5.1.2 Pericyte–macrophage interaction in the TME

Macrophages play a critical role in supporting tumor development by eliciting an immunosuppressive response and tissue remodeling. Several reports identify pericytes as the mediators responsible for tumor-associated macrophage (TAM) recruitment.

**FIGURE 2:**
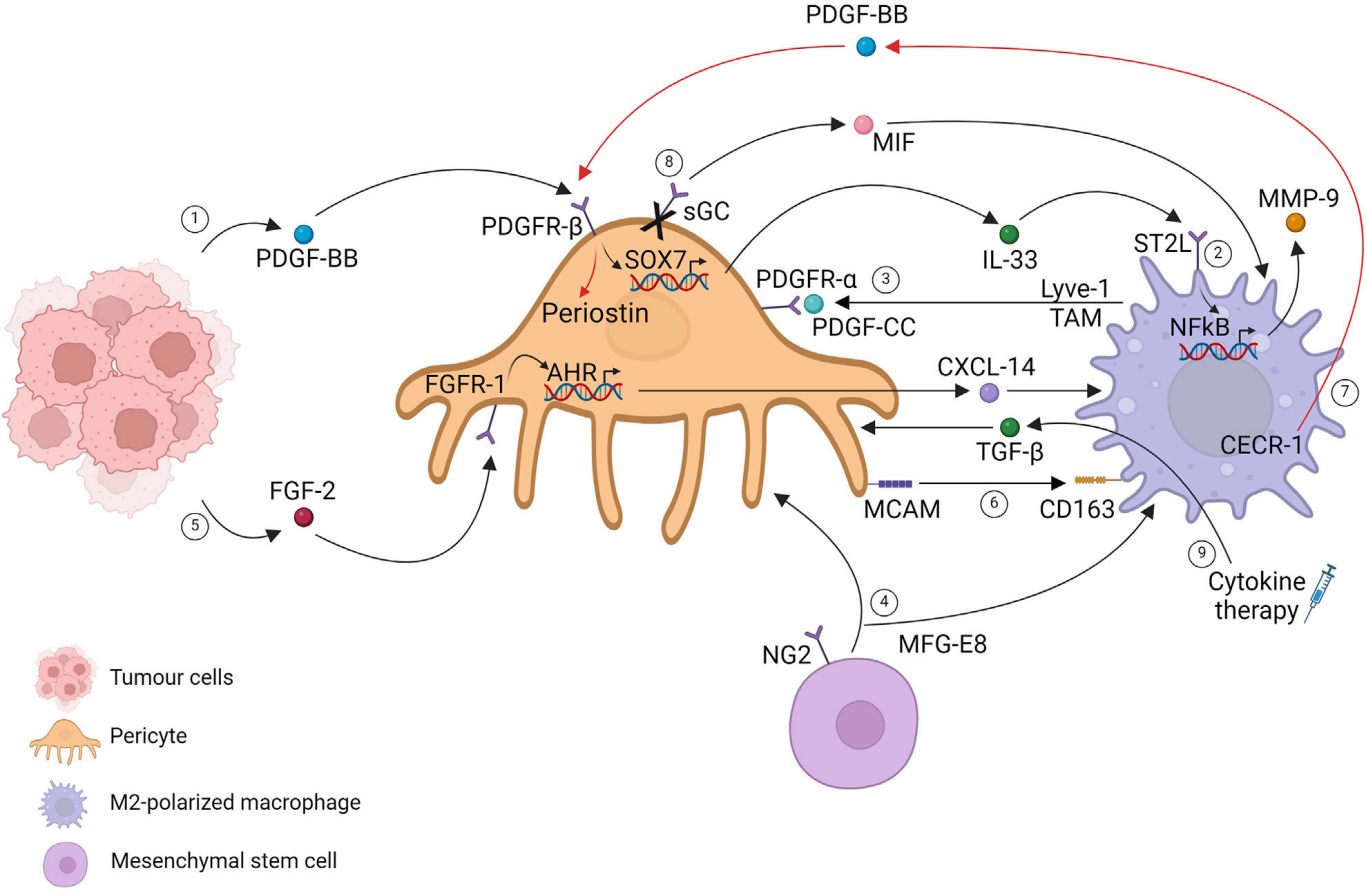
Pericytes and tumor-associated macrophages in the primary tumor mass. Created with BioRender.com.

Indeed, PDGF-BB-expressing tumors have a high potential to recruit TAM. PDGF-BB-stimulated pericytes and stromal fibroblasts induce IL-33 upregulation via the PDGFR-β pathway through a SOX7-dependent transcriptional mechanism. IL-33-triggered signaling stimulates the M2 phenotype, increasing metastasis formation through the production of several MMPs (Yang et al., 2016). The fact that this phenotype can be reverted by depleting TAM with clodronate liposomes highlights the crucial mediation of pericyte-recruited macrophages in invasiveness (Figure 2, path 1).

An update of this finding was conducted 2 years later, providing specific signaling pathways that regulate stromal cells. These pathways instruct immune cells within the primary tumor mass, facilitating cancer invasion through the release of molecules that mediate tissue remodeling. Considering a highly fibrotic tumor such as pancreatic ductal carcinoma, the authors show that IL-33 was mainly upregulated by cancer-associated fibroblasts (CAFs) and pericytes, driving M2 polarization mediated by the ST2 receptor. IL33 drives MMP9 production via an NF-kB-dependent mechanism, leading to laminin degradation, which can be a critical step for tumor cell extravasation and invasion (Figure 2, path 2) (Andersson et al., 2018).

PDGF-mediated host cell recruitment in the TME has also been described in the opposite direction: PDGF-CC expressing TAM crosstalks with PDGFR-α pericytes. Single-cell RNA sequencing in a spontaneous MMTV-PyMT murine model of breast cancer delineates a perivascular Lyve-1 TAM population characterized by the expression of a selective lymphatic vessel endothelial hyaluronic acid receptor. Lyve-1 TAM has been found near the vascular niche, especially on αSMA cells, which resemble a pericyte-like mesenchymal population that creates a pro-angiogenic niche. Although Lyve-1 TAM accounts for a small percentage of live cells within the tumor mass, their role is critical for tumor progression. Depletion of the subpopulation slows down tumor growth, accompanied by curtailments in αSMA pericyte-resembling cells. Lyve-1 TAMs control αSMA expansion in a PDGF-CC dependent manner, suggesting that pericyte proliferation in tumors is not only autonomous but tightly controlled by other cell types such as macrophages that consequently participate in vascular remodeling (Figure 2, path 3) (Opzoomer et al., 2021). Other pieces of evidence have demonstrated that mesenchymal stromal cells, which resemble pericytes, correlate with M2 macrophages. The authors demonstrated that the secreted glycoprotein MFG-E8 (lactadherin), involved in regulatory T-cell development and neovascularization, is released by pericytes (Motegi et al., 2011) and MSCs in melanoma. Melanoma cells implanted with MSC-resembling pericytes increase tumor proliferation and pericyte and M2 macrophage infiltration, suggesting vascular regulation mediated by MFG-E8 (Figure 2, path 4) (Yamada et al., 2016).

Another molecule investigated in TAM recruitment is CXCL14, which is involved in immune cell regulation, tumor reprogramming, and the metastatic process. The authors identified nasopharyngeal carcinoma (NPC) as a highly FGF2-expressing tumor type with predominant macrophage infiltration. FGF2 induces macrophage activation and M2 polarization, mediating selective pericyte secretion of CXCL14 via FGFR1. The described stromal cell interactions orchestrated by FGF2 tumoral cells have been shown to promote metastasis, identifying FGF2/pericytes/CXCL14/TAM as a promising targetable axis in NPC or more in general FGF2-expressing tumors (Figure 2, path 5) (Wang et al., 2022).

Pericytes and macrophages recognized by MCAM/CD163 gene pair expression have been identified as a potential prognostic immune signature co-expressed in glioblastoma (GBM) samples. The coupled cells and gene pairs could be used as an indicator for immunotherapy response rates. (Figure 2, path 6) (Zhang et al., 2022). Evidence has unveiled that tumor cells can shape pericytes via flectopodia. GBM cytoplasmic extension modifies pericyte contractile activity, shifting toward a macrophage-like phenotype and promoting immune suppression and tumor expansion (Caspani et al., 2014).

Pro-tumorigenic macrophages influence pericyte functions. In a model of intracranial B16F10 melanoma tumors, the specific ablation of NG2 proteoglycan in myeloid cells has been shown to reduce TAM recruitment with a direct decrease of pericyte–endothelial cell interaction, which is linked to N-cadherin loss (Yotsumoto et al., 2015). GBM microenvironment-M2-like macrophages have been demonstrated to promote pericyte recruitment and migration toward the extracellular adenosine deaminase protein CECR-1 via the PDGF-B–PDGFR-β axis downstream, stimulating periostin expression in pericytes with a proangiogenic activity on extracellular matrix components (Figure 2, path 7) (Zhu et al., 2017). Moreover, changes in TAM metabolism have been shown to affect tumor growth, angiogenesis, and metastasis. mTOR activation in TAM, by depleting one of its negative regulators (REDD1 depletion), installs a glucose competition with endothelial cells, promoting their quiescent state. Conversely, REDD1 upregulation under stress conditions inhibits mTOR signaling, which favors a deranged tumor vasculature. Notwithstanding that the metabolic competition between pericytes and TAMs is completely unexplored, these data highlight the critical role of metabolism in the TME cell–cell relationship, suggesting mTOR activation as anti-tumoral in TAM, suggesting a possible mechanism of mTOR targeting drug resistance (Wenes et al., 2016).

Drug interventions shed light on the intricate relationship between pericytes and macrophages in the tumoral context. Single-cell RNA-sequencing reveals a perturbation in endothelial cell and pericyte communication as a direct outcome of soluble guanylate cyclase (sGC) selective deletion in pericytes, guided by Notch signaling impairment. In addition, pericyte sGC deletion reveals an increased expression of macrophage inhibitory factor (MIF), which has been demonstrated to drive macrophage polarization from the M2 to M1 phenotype, suggesting its contribution to anti-tumoral inhibition (Figure 2, path 8) (Zhu et al., 2024). Few years ago, cytokine therapy was proposed to normalize the vascular bed with the goal of enhancing drug delivery. The restoration of the pericyte-contractile phenotype is induced by a specific lymphotoxin (LIGHT), which binds to the TNFSF14 receptor, a member of the TNF family, expressed on different cell types, mainly immune cells. TME-LIGHT treatment restores pericyte-contractile properties via TGFβ secretion by macrophages, normalizing the vascular bed. Low doses of intra-tumoral LIGHT trigger macrophage-TGFβ secretion in a Rho kinase-dependent manner, inducing a specific pericyte-contractile gene signature, decreasing vascular leakiness, and increasing tumoral perfusion (Figure 2, path 9) (Johansson-Percival et al., 2015).

#### 5.1.3 Pericyte–T cell interaction in the TME

Tumor-infiltrating lymphocytes affect tumor progression and can be divided into cytotoxic T cells, which hinder tumor growth while fighting against cancer, and regulatory T cells (Tregs), which represent the immunosuppressive response to prevent tumor clearance.

As described above for the pericyte–macrophage interaction, even pericyte crosstalk with T cells favors cancer progression by developing an immunosuppressive environment. Evidence in the literature, until the time of writing, described tumor-derived pericytes as mediators of immunosuppression by inhibiting T helper cell (Th cell) capabilities.

**FIGURE 3:**
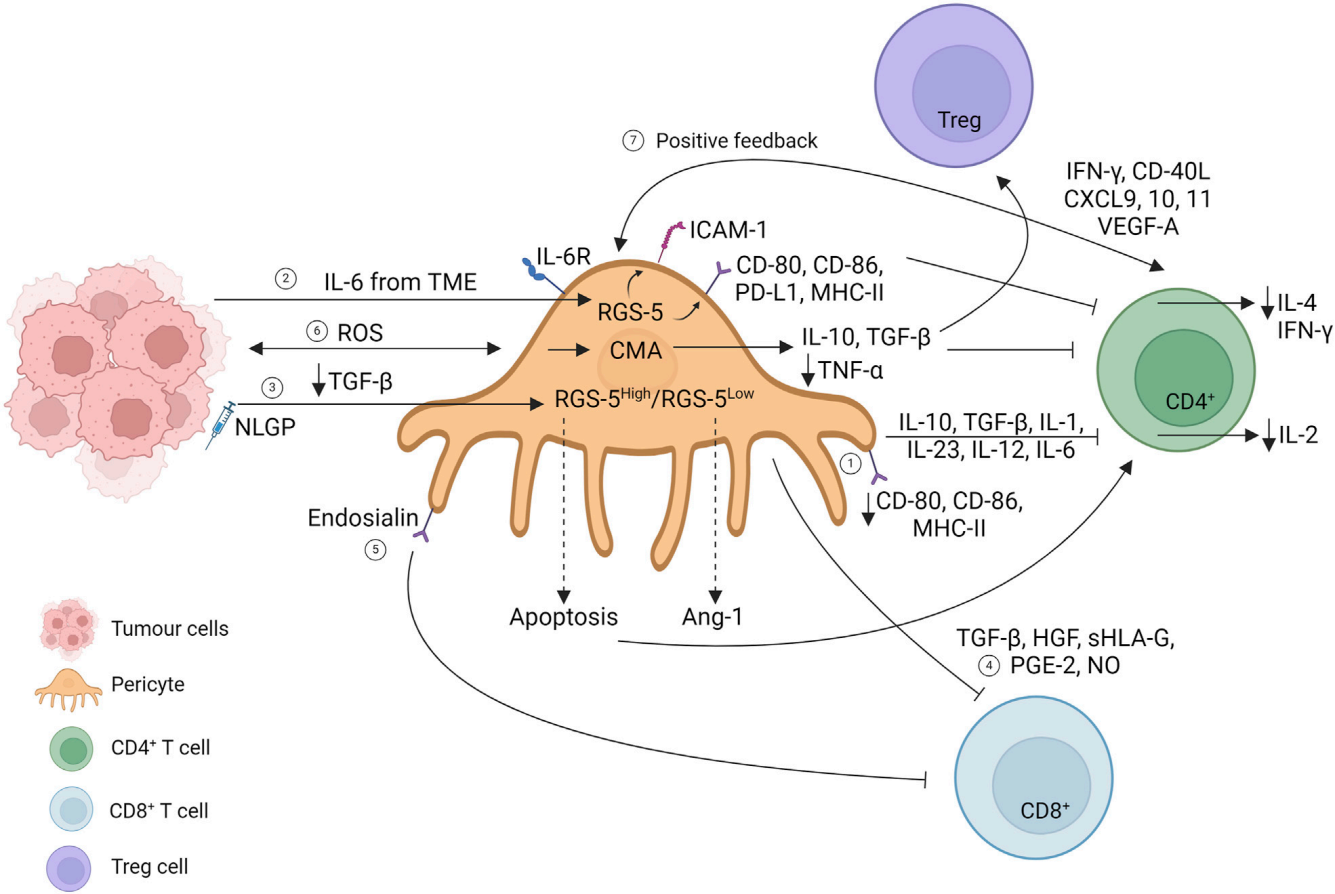
Interaction of pericytes and T cells in the primary tumor mass. Created with BioRender.com

The establishment of immune tolerance in GBM has been linked with brain pericyte immunosuppressive phenotype acquisition and the consequent failure to activate the T-cell response. Pericytes interacting with GBM acquire an anti-inflammatory phenotype, increasing the expression of IL-10, TGF-β, IL-1, IL-23, IL-12, and IL-6, accompanied by a decreased expression of co-stimulatory molecules such as CD80 and CD86 and a reduction in the expression levels of major histocompatibility complex class II (MHC-II) molecules, suggesting impaired antigen presentation ability by GBM-pericytes. Moreover, GBM negatively influences pericytes in activating T-cell responses. Isolated CD4^+^ T cells interacting with GBM-pericytes show decreased IL-2 secretion and proliferation, suggesting an impaired T-cell response due to pericyte acquisition of an anti-inflammatory phenotype promoting GBM proliferation (Figure 3, path 1) (Valdor et al., 2017).

Modulation of MHC expression seems to be a mechanism controversially related to T-cell activation. Indeed, in 2013, an article illustrated how tumor-derived B16 pericyte upregulated co-stimulatory molecules (CD80 and CD86), the co-inhibitory molecule (PD-L1), and MHC-II, which directly downregulate CD4^+^ T-cell proliferation and IL-4 and IFN-γ secretion. The overexpression of the marker gene RGS-5 and IL-6 stimulation of tumor-derived pericytes mediate CD4^+^ T-cell anergy *in vitro*, elicited by the upregulation of anergy-related factors (dgkα, erg2, and erg3). Moreover, RGS-5 and IL-6 upregulate ICAM-1 expression directly, promoting immune evasion, and indirectly upregulate the VCAM-1 adhesion molecule (Figure 3, path 2) (Bose et al., 2013). Immune evasion directed by RGS-5 tumor-derived pericytes has been further explored in B16F10 mass treated with a natural immunomodulator (neem leaf glycoprotein, NLGP) reported to normalize the vasculature in the TME (Banerjee et al., 2014). Exploring the NLGP mechanism for regulating vascular normalization, the authors show that NLGP treatment decreases both endothelial and RGS5^high^ pericyte cell numbers without affecting RGS5^low^ tumor-derived pericytes. In RGS5^high^ pericytes, elevated TGF-β levels in the TME promote RGS-5 interaction with the cytoplasmic mediator SMAD2/3. The RGS5-SMAD2/3 complex translocates in the nucleus, promoting pericyte proliferation with consequent aberrant vascularization and CD4^+^ T-cell anergy. NLGP treatment suppresses TGF-β levels, preventing RGS-5 nuclear translocation, leading to apoptotic RGS5^high^ pericyte subpopulation, and restoring T-cell function. Meanwhile, NLGP treatment also affects RGS5^low^ tumor-derived pericytes, which upregulate the Ang-1 molecule, restoring the endothelial–pericyte interaction (Figure 3, path 3) (Dasgupta et al., 2022).

Glioma tumor-derived pericytes have been shown to express MSC markers (CD90, CD248, and PDGFR-β), and their expression correlates with a decrease in leukocyte and CD8 T-cell infiltration. Moreover, TGF-β secretion together with HGF, sHLA-G, PGE2, and NO expression has been shown to impact the proliferation of PBMC cultures, suggesting the local immunosuppressive abilities acquired by MSC-like tumor-derived pericytes (Figure 3, path 4) (Ochs et al., 2013).

A reduction in CD8 renal cell carcinoma infiltration has been correlated with a selective tumor-derived pericyte population that overexpresses endosialin, a transmembrane glycoprotein promoting tumor-derived pericyte proliferation and migration. Tumor-derived pericytes are a heterogeneous population, and specific marker expressions have been identified in T-cell interactions (Bose et al., 2013, Dasgupta et al., 2022). Endosialin knockout (EN^KO^) pericytes have been shown to increase T-cell infiltration *in vitro*, suggesting that endosialin expression could impede cytotoxic infiltration. This situation can be reversed by combining anti-endosialin treatment with anti-PD1 therapy (Figure 3, path 5) (Lu et al., 2023).

To increase the complexity of the scenario, pericyte-chaperone-mediated autophagy (CMA) has been proposed to control tumor progression. CMA refers to the selective degradation of targeted proteins that upregulates LAMP-2A expression at the lysosomal membrane. Although maintaining strict regulation of CMA is necessary for homeostasis, the hyper-activation of tumors correlates with tumor progression. Glioma–pericyte interaction generates ROS production, which consequently upregulates LAMP-2A expression on pericytes, which mediates the acquisition of an immunosuppressive phenotype in these cells, increasing IL-10 and TGF-β expression and downregulating TNF-α. Isolated CD4^+^ T cells from glioma-mice-lymph nodes have been shown to upregulate inhibitory molecules, such as CTLA-4 and PD-1, and decrease IL-2 production compared to CD4^+^ T cells isolated from engrafted tumors with LAMP-2A^KO^ pericytes (Valdor et al., 2019).

Some years later, an in-depth RNA sequencing analysis revealed new networks under CMA hyperactivation in GBM-derived pericytes. LAMP-2A^KO^ pericytes upregulate inflammatory responses and phagocytosis as anti-tumoral responses, thereby hindering immune cell evasion under abnormal CMA regulation. Moreover, the secretome of LAMP-2A^KO^ pericytes reveals a decrease in inflammatory proteins such as gelsolin, periostin, and osteopontin, accompanied by an increase in anti-tumoral proteins such as lumican and vitamin D. CMA ablation in pericytes results in increased CD4^+^ T-cell activation with upregulation of PD-1 and CTLA-4, decreased Treg infiltration, and macrophage activation with the CD68 marker, suggesting an elicited immune response to hinder tumor cell growth (Figure 3, path 6) (Molina et al., 2022).

Pericytes do not solely play an immunosuppressive role, but a recent article shows positive regulatory feedback among tumor-derived pericytes and Th cells. CD4^+^ T cell KO in breast cancer murine models (EO771, 4T1 tumors) has been linked with a decrease in pericyte coverage accompanied by an increase in metastatic potential. EO771–pericyte depletion, under diphtheria toxin treatment, decreases CD4^+^ T cell, TAM, and dendritic cell infiltration, while, on the other hand, Th cell transfer promotes the opposite effects, elucidating a positive feedback mechanism between pericytes and Th cells. The use of the immune checkpoint blockade promotes vascular normalization, leading to an increase in pericyte coverage, an increase in CD4^+^ T cells (specifically Teff while diminishing Treg cells) and dendritic cells, and a decrease in neutrophil population, correlating with a decrease in metastasis number. The new finding that highlights Th cell loops with vascular normalization has been shown to be mediated by IFN-γ and CD40L expression linked with concomitant chemokine expression (CXCL9, CXCL10, and CXCL11) and VEGFA endothelial expression (Figure 3, path 7) (Tian et al., 2017). The increase in pericyte coverage that correlates with an increase in immune infiltration has also been shown by Yang et al., who evaluated agonists to stimulate the IFN gene pathway (STING) to normalize the tumor vasculature. STING agonists have been shown to increase pericyte coverage and leukocyte vascular adhesion molecules such as ICAM and VCAM, promoting CD8^+^ T-cell infiltration while simultaneously increasing M1 macrophage-promoting genes (Yang et al., 2019).

#### 5.1.4 Pericyte–MDSC interaction in the TME

Myeloid-derived suppressor cells (MDSCs) counteract the other immune cells defending tumor cells from T cell, macrophage, and dendritic cell anti-tumor reactivity. They retain immunosuppressive roles to maintain tumor supremacy and cancer progression. MDSC represents a heterogeneous myeloid-derived cell population that can express a granulocytic or monocytic phenotype, respectively, characterized by Ly6G^+^ or Ly6C^+^ expression.

Pericyte–MDSC crosstalk in tumors has been described in 2015 using melanoma and LLC animal models with pericyte deficiency (PDGFR-β^ret/ret^ mice), drawing a negative correlation between pericyte coverage and MDSC (GR1^+^/Cd11b^+^) infiltration specifically in tumors associated with an increase in serum IL-6. IL-6 silencing normalizes the rate of B16 circulating cells, which can be associated with a reduced hypoxic rate in B16 normalized pericyte coverage (Hong et al., 2015). Moreover, in a breast cancer cohort, a subtype of patients identified with high pericyte coverage and a decrease in MDSC accumulation has been identified with a better prognosis, delineating the powerful correlation between pericytes and MDSC trafficking in tumors (Hong et al., 2015). Recently, a pericyte population that resembles stem cells has been identified (CD45^−^EPCAM^-^CD29^+^CD106^+^CD24^+^CD44^+^). These pericytes can instruct MDSCs to generate an immunosuppressive environment in a pancreatic tumor model. The co-injection of pancreatic tumoral cells with pericyte-like stem cells increases tumor proliferation and CD45^+^ infiltration, mainly represented by Ly6G^+^ MDSC differentiation, while macrophages and dendritic cells have been shown to be reduced. Furthermore, the use of an anti-PD1 antibody has been shown to activate the cytotoxic response only when tumoral cells are not co-injected with pericyte stem cells, indicating that this pericyte population can be targeted to overcome immunotherapy resistance (Wu et al., 2023).

#### 5.1.5 Pericytes, NK-cells, and B cells in the TME

Unlike the considerable research conducted on the interaction between tumor-derived pericytes and previously mentioned immune cell types, there is a noticeable lack of publications exploring their correlation with natural killer (NK) cells, dendritic cells, and B cells.

NK cells are deputed in the anti-tumor response, activating a cytotoxic response against target cells, and usually infiltrate hypoxic environments. In this regard, a work published in 2017 explored the selective HIF-1α deletion in NK cells (HIF-1α-NK^KO^). HIF-1α-NK^KO^ mice engrafted with colon cancer cells (MC38) or LLC showed decreased tumor growth, even as assessed by an increase in cell death (caspase-3 staining), accompanied by a reduction in pericyte coverage and an increase in Glut-1, which correlates with the hypoxic state. Notwithstanding the decreased tumor proliferation, HIF-1α-NK^KO^ tumors did not show metastatic reduction, suggesting that vessel abnormalities caused by the NK hypoxic state reflect leakier vessels that can be compassable by circulating tumoral cells (Krzywinska et al., 2017).

The only evidence that connects pericytes with B cells dates back to 2010. Authors exploring primary central nervous system lymphoma (PCNL) found major CD8 T-cell infiltration in vascular areas expressing CXCL9, which has been shown to be mainly expressed by pericytes and macrophages. Other than CXCL9, CXCL12 shows an increased expression pattern in PCNL, and *in vitro* studies reveal that together with CXCL9, it forms a heterocomplex. The two cytokines synergize together, recruiting CXCR3^+^/CXCR4^+^/CD8^+^ and malignant CXCR4-dependent B-cell migration (Venetz et al., 2010).

#### 5.1.6 Pericyte and neutrophil crosstalk: gap in knowledge about cancer

Neutrophils represent a major population of white blood cells and are divided into pro-tumorigenic and anti-tumorigenic neutrophils, similar to macrophage classification.

Three-dimensional real-time imaging shows that neutrophils migrate toward pericyte gaps in an ICAM- and KC (keratinocyte-derived chemokine)-dependent manner, expressing ligands such as Mac-1 and LFA-1. Pericyte shape is influenced by pro-inflammatory cytokines such as TNF-α and IL-1β that bind to receptors present on pericytes, leading to gap enlargement that facilitates neutrophil transmigration (Proebstl et al., 2012). Two-photon microscopy demonstrates how NG2 pericytes can instruct extravasated leukocytes and macrophages, improving cell motility that results in faster and more directional immune cell migration. Pro-inflammatory stimuli, such as TNF and DAMP (damage-associated molecular pattern), increase mainly MIF (macrophage migratory inhibitory pattern) pericyte secretion other than CCL2 and CXCL5, together with ICAM upregulation, which mediates leukocyte orientation through CXCL8 and TLR4 and TLR9 expression in neutrophils (Stark et al., 2013). Pericytes can promote neutrophil activation via the IL-17-IL17RA pathway. Following IL-17 stimulation, pericyte activation promotes neutrophil secretion of pro-inflammatory molecules (IL-1α, IL-1β, TNF, and IL-8), improves neutrophil survival through the secretion of G-CSF and GM-CSF, and enhances neutrophil phagocytosis (Liu et al., 2016). RNA sequencing analysis provides a panel of upregulated chemokines involved in pericyte recruitment when *in vitro* pericytes are co-cultured with macrophages upon *Streptococcus* pneumoniae stimulation. Pericyte expresses a higher amount of neutrophil-attracting chemokines, among which CXCL8-derived pericytes have been shown to induce neutrophil *in vitro* transmigration independently of the endothelial barrier (Gil et al., 2022). Another piece of evidence confirms IL-8 pericyte secretion in the mediation of neutrophil migration, showing that inflammatory stimuli increase their adhesion, which is a process mediated by MMP-2/9 (Pieper et al., 2013).

All the above-described interactions between pericytes and neutrophils show the relevance of their crosstalk in the regulation of immune responses but highlight a gap in knowledge regarding their communication in the tumor microenvironment. The only evidence to our knowledge that correlates pericytes and neutrophils in cancer (tumor-associated neutrophils, TANs) indicates that neutrophils are the main cellular source of pro-angiogenic MMP9, quantitatively surpassing TAMs. MMP-9 increased release has been demonstrated in both isolating neutrophils and macrophages from healthy tissue and investigating *in vivo* human prostate cancer xenografts and Lewis lung carcinoma (LLC) mouse models. Tumors enriched in MMP-9-delivering TAN are characterized by enlarged vessels (11–20 µm in diameter), which are partially covered by pericytes, suggesting a possible way for tumor dissemination. MMP knockout mice show a decrease in pericyte coverage, suggesting a correlation between neutrophils and their angiogenic counterparts (Deryugina et al., 2014).

The information provided above emphasizes the importance of investigating pericytes as a crucial stromal component of the TME. This involves understanding their communication with immune cells and how their interactions are influenced by exposure to malignant cells, which can subsequently impact their interactions with other TME cells. Moreover, within the intricate dynamics of the TME, molecules from non-tumor cells can also play a role in facilitating cancer cell extravasation.

### 5.2 Tumor reprogramming and epithelial-to-pericyte transition

Cancer cells from the primary mass, under the influence of TME remodeling and the formation of abundant vessels unable to sustain nutrient and oxygen demand, move toward a distant site where they colonize and generate a secondary metastatic mass. A cancer cell that leaves the primary mass is named a circulating tumoral cell (CTC). CTCs intravasate into the primary mass vessels, move through the bloodstream, and extravasate from vessels into new distant tissues. One of the main orchestrators for the intravasation of future CTCs is the neutrophil. Indeed, single-cell transcriptomic studies suggest that CTCs originate from clusters with neutrophils via upregulation of VCAM1 and ICAM1, capitalizing on the innate abilities of these cells to facilitate their migration and intravasation. Furthermore, interleukin 8 (IL8 or CXCL8) produced by CTCs promotes the adhesion of neutrophil/CTC clusters to the endothelium for extravasation at the pre-metastatic niche (Figure 1) (Guo and Oliver, 2019; Di Russo et al., 2024). Based on the fact that pericytes play an active role in the transmigration of immune cells, including neutrophils (Stark et al., 2013), we suppose that they may coordinate the neutrophil/CTC cluster intravasation/extravasation, a field of research that remains largely unexplored but holds significant promise.

This complex series of events is controlled by a process called epithelial-to-mesenchymal transition (EMT), during which the epithelial cancer cells can undergo reduction of cell–cell adhesions, reorganization of the actin cytoskeleton, and transformation into spindle-shaped mesenchymal cells with heightened migratory and invasive capabilities. Classically, during EMT, epithelial markers such as adherent junction protein E-cadherin and tight junction proteins like claudins are downregulated, while mesenchymal markers such as adhesion protein N-cadherin, intermediate filament protein vimentin, fibroblast-specific protein 1 (FSP1), and smooth muscle α-actin (αSMA) are upregulated (Pastushenko and Blanpain, 2019).

Despite the huge amount of data present in the literature, the EMT process is still not fully understood. Indeed, in aggressive breast cancer, the higher expression of E-cadherin often correlates with a more invasive and metastatic phenotype (Fulga et al., 2015). Moreover, lineage-tracing experiments have revealed that the majority of metastatic cancer cells do not activate the promoters of FSP1 and vimentin, which are recognized as *bona fide* mesenchymal markers. Furthermore, inhibiting EMT does not impact the formation of spontaneous lung metastases, indicating that EMT is not essential for metastasis (Fischer et al., 2015).

In this already complex and confounding scenario, the mesenchymal products generated by EMT often express multiple pericyte markers and associate with and stabilize blood vessels to fuel tumor growth, thus phenotypically and functionally resembling pericytes. This additional process is called epithelial-to-pericyte transition (EPT), which defines the ability of cancer cells to become pericyte-like cells.

One of the first pieces of evidence of EPT was reported by Cheng et al., in 2013. Their study demonstrated that, *in vivo*, the cell lineage tracing with constitutive and lineage-specific fluorescent reporters showed that glioma stem cells (GSCs) generate the majority of vascular pericytes in glioblastoma (Cheng et al., 2013). By tracking epithelial cancer cells that underwent inducible or spontaneous EMT in various tumor transplantation models, it was possible to demonstrate that the majority of EMT cancer cells were specifically located in the perivascular space and closely associated with blood vessels. EMT markedly activated multiple pericyte markers in carcinoma cells, in particular PDGFR-β and N-cadherin, which enabled EMT cells to be chemoattracted toward and physically interact with the endothelium (Shenoy et al., 2016).

Other pieces of evidence in glioblastoma support the existence of EPT. For example, conditioned medium from glioblastoma increased the proliferation and migration of glioblastoma-derived mesenchymal stem cells (gb-MSCs) and could induce their differentiation into pericytes. Glioblastoma secretes angiogenic factors, and gb-MSCs cultured in a malignant glioblastoma-conditioned medium exhibited a greater formation of tube-like structures. Additionally, these cells adhered to tube-like vessels formed by human umbilical vein endothelial cells (HUVECs) (Yi et al., 2018). From a pharmacological point of view, the blood–tumor barrier (BTB) is a major obstacle for drug delivery to malignant brain tumors such as glioblastoma, and targeting GSC-derived pericytes specifically disrupts the BTB and enhances drug effusion into brain tumors (Zhou et al., 2017).

In a very recent work, Huang et al. demonstrated that CD44^+^ cancer stem cell (CSC)-derived pericyte-like cells, referred to as Cd-pericytes, display remarkable potential for trans-endothelial migration, facilitating effective intravasation and extravasation through GPR124-mediated Wnt7-β-catenin activation. These Cd-pericytes also possess the ability to dedifferentiate into tumorigenic CSCs, initiating brain metastases. Consequently, targeting Cd-pericytes, GPR124, or Wnt7-β-catenin signaling significantly inhibits tumor metastasis (Huang et al., 2023).

To provide a comprehensive overview, it is crucial to acknowledge the capability of pericytes to differentiate into fibroblasts, a phenomenon termed pericyte-to-fibroblast transition (PFT), as mentioned previously. In the cancer context, gain- and loss-of-function experiments demonstrate that PDGF-BB–PDGFRβ signaling promotes PFT both *in vitro* and *in vivo* different human tumors, which significantly contributes to tumor invasion and metastasis (Hosaka et al., 2016). Interestingly, when pericytes are cultured on soft polyacrylamide (PA) gels resembling the pliability of healthy tissue, they maintain their original characteristics and actions. However, when placed on stiff PA gels resembling the rigidity of tumor tissue, pericytes tend to undergo PFT and exhibit increased mobility and invasiveness (Feng et al., 2021).

At present, it is evident that cancer stem cell-derived pericytes are able to interact with endothelial cells to coordinate the intravasation/extravasation, but the relation between cancer stem cell-derived pericytes and the inflammatory milieu still remains unexplored.

### 5.3 Pericyte–inflammatory cell crosstalk in the pre-metastatic niche

Tumor metastasis is the leading cause of most cancer-related deaths. An important issue is that some tumors show a predisposition to metastasize to selected organs, probably influenced by the interaction with host factors (van Zijl et al., 2011). The concept that the colonization of the secondary site is not a matter of chance was described by Stephen Paget in 1889 with the “seed and soil” theory, which proposed that the growth of tumor cells (the seed) occurs in the distant organ (the soil), providing a proper environment (Paget, 1989). However, the specific properties of tissues that influence their colonization by tumor cells remain to be elucidated.

Increasing evidence shows that the primary tumor supports metastasis formation by inducing the construction of a receptive microenvironment in secondary organs, termed the pre-metastatic niche, which improves metastatic cell survival and proliferation (Liu and Cao, 2016). The pre-metastatic niche consists of a complex microenvironment that contains various types of cells, including vascular and stromal cells, immune cells, extracellular matrix proteins, and tumor-produced molecules (Paiva et al., 2018). Primary tumors are believed to produce and liberate exosomes into the bloodstream that induce changes in the microenvironment of distant organs. Cancer-derived exosomes exert an influence on the pre-metastatic microenvironment by affecting several cell types (Paiva et al., 2018).

Pericytes making up vessels in the pre-metastatic niche are altered in terms of reduced numbers and weaker connections with endothelial cells and the basement membrane, resulting in leaky vessels with increased permeability, thereby facilitating cancer cell extravasation (Jiang et al., 2023). Guarino et al. (2021) demonstrated that tumor-derived extracellular vesicles (t-EVs) induce pathological angiogenesis. This is supported by decreased pericyte coverage and increased co-localization of vascular endothelial growth factor 2 (VEGFR2) with the endothelium, achieved through TRPV4 downregulation-mediated activation of Rho/Rho kinase/YAP/VEGFR2 pathways (Guarino et al., 2021). In a breast cancer brain metastasis model, high permeability of the BTB is associated with an increase in desmin^+^ and a decrease in CD13^+^ pericyte coverage, thus suggesting that altered prevalence of pericyte subpopulations can influence the BTB permeability (Lyle et al., 2016).

In addition to the direct involvement of pericytes, there is evidence of a pericyte-like function in different cellular types in the pre-metastatic niche. Disseminated tumor cells can exploit pericyte locations to migrate and extravasate into the target organ. Lugassy et al. (2024) described the migration capabilities of melanoma cells along the outside of vessels, a mechanism termed “extravascular migratory metastasis,” taking a pericyte-like position (Lugassy et al., 2024). Disseminated cancer cells further employ cell adhesion molecule L1 (L1CAM) in pericytes to mediate their adhesion and spreading along the basement membrane of the vasculature; this displaces resident pericytes and facilitates their spread on capillaries through the activation of L1CAM-YAP signaling (Er et al., 2018).

As mentioned in the previous paragraph, CD44^+^ lung CSCs generate the majority of pericytes in the perivascular niches of lung adenocarcinoma. These CSC-derived pericyte-like cells (Cd-pericytes) exhibit enhanced trans-endothelial migration (TEM) capacity through G-protein-coupled receptor 124 (GPR124), allowing them to intravasate into the vessel lumina, survive in the bloodstream, and extravasate into the metastatic site. Upon arrival at the brain parenchyma, Cd-pericytes are able to de-differentiate into tumorigenic CSCs, facilitating metastasis formation (Huang et al., 2023). Moreover, MSCs can display a pericyte-like function in the metastatic site. Melanoma cancer cells co-expressing CD146 and Sdf-1/CXCL12-CXCR4 signaling interact with local MSC-derived pericytes, which regulate their extravasation to murine bone marrow and liver (Correa et al., 2016).

Pericytes, in addition to their role as perivascular cells, also act as effector cells involved in the establishment of the pre-metastatic microenvironment. Tumor-derived factors secreted by cancer cells induce the loss of traditional surface markers in pulmonary pericytes by increasing the expression of the pluripotency gene *Klf4*, followed by enhanced extracellular matrix synthesis that creates a pro-metastatic fibronectin-abundant environment (Murgai et al., 2017). Colorectal cancer-secreted factors (CCSFs) induce a decrease in cellular extensions and phosphorylated myosin light chain (p-MLC) levels in pericytes that are associated with a relaxed pericyte phenotype, resulting in gaps in vessel coverage. CCSFs also inhibit pericyte activities of degrading or depositing collagen, leading to reduced collagen density within the microenvironment (Nairon et al., 2022). A recent study shows that pancreatic ductal adenocarcinoma (PDAC)-derived exosomes induce an ectopic α-smooth muscle actin (αSMA) expression in pericytes that correlates with intratumoral hypoxia and vascular leakiness. Moreover, αSMA + pericytes exhibit an immunomodulatory phenotype, as evidenced by the increased expression of CD80, CD86, HLA-DRA, and CD274 immunoregulatory molecules (Natarajan et al., 2022).

To reach the target site, inflammatory cells must overcome several physical barriers, including the endothelium, the vascular basement membrane, and the pericyte coverage (Armulik et al., 2011). It is therefore reasonable that the presence of leaky vessels with increased permeability facilitates immune cell extravasation. Some pieces of evidence indeed demonstrate that sites characterized by gaps between pericytes are preferentially used by inflammatory cells to overcome the vessel wall (Wang et al., 2006; Voisin et al., 2010).

A recent study assessing the transcriptional alteration using single-cell RNA sequencing on brain metastasis shows that the extent of pericyte coverage negatively correlates with immune cell infiltration. Brain metastases were shown to be enriched in clusters of pericytes that interfere with extracellular matrix synthesis, mobility, and vascularization. Mural cell depletion has been found to correlate with an amount of total CD45^+^ cell infiltration and an increase in CD3^+^ cells, which can be possibly mediated by the downregulation of claudin-5, as shown in pericyte-depleted tumors. Moreover, immune checkpoint molecules like CD276 have been found upregulated in both endothelial and mural cells in brain metastasis, highlighting the tumor-associated vasculature as a crucial player in immune regulation (Bejarano et al., 2024).

Beyond the physical matter of vessel coverage, a lot of studies have illustrated the direct crosstalk between pericytes and immune cells that occurs in the primary tumor site, but the interaction between these two cell types in the pre-metastatic microenvironment remains to be elucidated. The proof of interaction between pericytes and immune cells was obtained from Wang et al., who compared normal, tumor, and metastasis samples of lung adenocarcinoma to explore different cellular landscapes. The cell–cell communication analysis of metastasis samples reveals an increase in pericytes in metastatic tissue, where they interact with B cells, cytotoxic T cells, dendritic cells, endothelial cells, epithelial cells, M1 macrophage, memory T cells, regulatory T cells, monocytes, and NK cells *via* the MDK-NCL pathway. This interaction represents an important hallmark in the transformation of the tumor microenvironment and indicates the activation of cancer-associated fibroblast (CAF)-mediated downstream pathways that facilitate tumor invasion (Wang et al., 2023). Only a few other studies explored potential pericyte–immune cell interactions in the metastatic microenvironment. It has been demonstrated that distant primary tumors induce matrix metalloproteinase 9 (MMP9) release in endothelial cells and macrophages *via* VEGFR-1/Flt-1 tyrosine kinase in the pre-metastatic lung and that it significantly promotes lung metastasis (Hiratsuka et al., 2002). Moreover, Yan et al. (2010) showed that Gr-1+CD11b+ MDSCs are increased in the lung before metastatic cell arrival and promote decreased pericyte coverage and aberrant vasculature formation through the production of MMP9 (Yan et al., 2010).

Further investigations are warranted to elucidate the reciprocal influence between pericytes and immune cells in the formation of the pre-metastatic niche. This area of research holds great potential for uncovering novel therapeutic targets and strategies aimed at disrupting the metastatic process in cancer.

## 6 Pericytes in cancer therapy

Conventional cancer therapy approaches have focused primarily on fighting tumors by completely cutting off their blood supply, targeting directly PDGF and/or VEGF.

However, anti-angiogenic drugs showed limited efficacy in clinics for several types of solid tumors since decreased pericyte coverage in tumor vasculature results in leaky vessels with increased permeability, thereby facilitating cancer cell intravasation and metastasis formation (Meng et al., 2015). Indeed, the presence of leaky vessels in tumors can result in reduced blood flow, leading to greater difficulty for antineoplastic drugs to effectively reach the tumor mass.

For this reason, new emerging therapies involve vascular normalization, consisting of remodeling tumor vessels to re-establish their structure and function, thus improving tumor perfusion to normalize the delivery rate of oxygen and reduce hypoxia, preventing cancer cell intravasation, and conveying therapies (Majidpoor and Mortezaee, 2021; Yang et al., 2021; Choi et al., 2023). In tumors with poor pericyte coverage, maintaining vessel integrity with pericyte-targeted vascular normalization therapies could prevent metastasis and improve treatment outcomes.

### 6.1 Anti-angiogenic drugs targeting VEGF signaling and their impact on vascular normalization

In 1993, Napoleone Ferrara’s group demonstrated that treatment with an anti-VEGF monoclonal antibody decreases vascular density and inhibits tumor growth in nude mice bearing xenografts of rhabdomyosarcoma, glioblastoma multiforme, and leiomyosarcoma (Kim et al., 1993). These results were confirmed in murine models of colorectal cancer, where the administration of a VEGF monoclonal antibody led to a dose- and time-dependent growth delay of subcutaneous xenografts and an accompanying reduction in the number and size of liver metastasis (Warren et al., 1995). However, despite the promising preclinical results, the effects of anti-VEGF monotherapy in clinical trials have not always been promising.

The first available anti-angiogenic therapy was bevacizumab, a macromolecular human monoclonal antibody that prevents the activation of VEGF signaling pathways by binding to soluble VEGF isoforms, thus preventing the interaction of VEGF with its receptor (Liu et al., 2023). Pericytes are believed to have an active role in the efficacy of bevacizumab treatment in colorectal cancer. Chondroitin sulfate proteoglycan 4 (CSPG4) is a cell surface proteoglycan released from pericytes to influence tumor angiogenesis. A recent study shows that the CSPG4-positive group had a better response rate to bevacizumab treatment, as well as longer progression-free survival (PSF) or overall survival (OS) (Besler et al., 2022).

Bevacizumab monotherapy in metastatic melanoma patients demonstrated promising clinical efficacy, showing disease control in 31% of patients (Schuster et al., 2012). In high-grade gliomas instead, early phase II clinical trials with bevacizumab showed promising results, but these results have not been confirmed in Phase III trials (Khasraw et al., 2014). Moreover, bevacizumab maintenance monotherapy during chemotherapy-free intervals (CFI) in metastatic colorectal cancer did not improve tumor control duration, CFI duration, PSF, or OS (Aparicio et al., 2018).

If clinical trials of anti-VEGF monotherapy have generally had negative results in patients with solid tumors, combination approaches of anti-VEGF therapy with conventional chemotherapy have demonstrated improved survival in cancer patients compared with chemotherapy alone. Disruption of the pro-angiogenic VEGF–VEGFR interaction normalizes the tumor vasculature, promoting the efficacy of chemotherapeutic agents by increasing their delivery into the tumor mass (Shrimali et al., 2010). For example, the combination of bevacizumab with systemic chemotherapy significantly improves PSF and OS in large, randomized phase III clinical trials compared to systemic chemotherapy alone (Goel et al., 2011). In 2004, the FDA approved bevacizumab in combination with intravenous 5-fluorouracil-based chemotherapy for first- or second-line treatment of patients with metastatic colorectal cancer. During the years following the first indication, bevacizumab was approved for various other cancers in combination with chemotherapy (Majidpoor and Mortezae, 2021; Liu et al., 2023).

Tumor relapse after bevacizumab therapy could also be bypassed with the administration of anti-idiotype (Id) antibodies to maintain stable levels of VEGF-binding antibodies. Sanches et al. (2016) demonstrated that mice immunized with an anti-bevacizumab idiotype mAb have reduced B16-F10 tumor growth, with more extensive necrotic areas, reduced CD31-positive vascular density, and decreased infiltration of CD68-positive cells (Sanches et al., 2016).

Vaccine-based therapy against VEGFR showed promising results. A phase II trial reported that gemcitabine in combination with elpamotide, a peptide vaccine containing HLA-A*24:02-restricted epitope peptide of VEGFR-2, improves the median OS of patients with advanced or recurrent biliary tract cancer. Moreover, it has been reported that patients who developed injection site reactions, probably signs of delayed hypersensitivity provoked by peptide-reactive T cell sensitization, have a significantly extended median OS compared to patients without injection site reactions (Matsuyama et al., 2015).

In some preclinical studies, anti-VEGF therapy improved vascular pericyte coverage, resulting in a reduction in metastatic cell intravasation, tumor hypoxia, and interstitial fluid pressure and increased delivery, as well as efficacy of cancer therapies (Goel et al., 2011). Shrimali et al. (2010) showed that the vessel normalization induced by VEGF/VEGFR-2 axis inhibition increases extravasation of adoptively transferred T cells into the tumor and improves adoptive cell transfer (ACT)-based immunotherapy in the B16 melanoma model (Shrimali et al., 2010).

Normalizing blood vessels facilitates immune cell penetration into tumors. Combining vascular normalization with immunotherapy may be a good approach to improving treatment outcomes (Quian et al., 2023). Several anti-angiogenic drugs have been proved to have an effect on normalizing blood vessels (Wilhelm et al., 2011; Sharma et al., 2013; Hillman et al., 2014; Liu et al., 2021). Immunotherapeutic drugs even show vascular normalization effects; it has been reported that the production of IFN-γ induced by immune checkpoint inhibitors (ICIs) in CD4+/CD8+ T cells improves vascular normalization by stimulating the accumulation of pericytes in blood vessels and increasing vasculature pericyte coverage. These findings suggest that vessel perfusion deflects the activation of T cell immunity against tumors, and it could be considered an indicator for predicting immune checkpoint blockade responsiveness. Since anti-angiogenic treatment has a narrow effect on vascular normalization, the association between anti-angiogenic therapy and ICIs can expand the normalization window (Zheng et al., 2018; Liu et al., 2021).

Dr. Rakesh K. Jain introduced the idea that the use of a proper dose of anti-angiogenic agents could reduce vascular permeability and interstitial fluid pressure and improve tumor vessel perfusion to repristinate the structure and function of tumor vessels (Jain, 2005). In this view, it has been demonstrated that lower doses of anti-VEGF receptor 2 (VEGFR2) antibodies, but not high anti-angiogenic doses, result in a more homogeneous distribution of functional tumor vessels. Lower doses are furthermore superior in enabling CD4^+^ and CD8^+^ T-cell tumor infiltration and changing tumor-associated macrophages from an immune inhibitory M2-like polarization toward an immune stimulatory M1-like phenotype. Based on this mechanism, lower-dose anti-VEGFR2 therapy with a whole cancer cell vaccine therapy that induces T-cell activation showed enhanced anticancer efficacy in murine breast cancer models (Huang et al., 2012).

Together, these findings indicate that the vascular-normalization impact of anti-VEGFR drugs reprograms the TME from immunosuppression toward an anti-cancer effect and improves other cancer therapies.

### 6.2 Vascular normalization via anti-PDGF agents

Inhibition of PDGFRβ signaling significantly affects tumor progression and angiogenesis, depending on PDGF-BB expression, compromising pericyte coverage and tumor vascularization (Tsioumpekou et al., 2020).

Targeting PDGFRβ with pharmacologic intervention has shown significant results in the treatment of several solid tumors. It has been demonstrated that imatinib, a receptor tyrosine kinase inhibitor (TKI), compromises lymphoma cell growth in both human xenograft and murine allograft models. Specifically, imatinib induces apoptosis of tumor-associated PDGFRβ1 pericytes, followed by disruption of tumor vasculature integrity (Ruan et al., 2013). Depletion of PDGFRβ+ pericytes also induces apoptosis of CD31^+^ vascular ECs and loss of perivascular integrity *in vivo* (Chute and Himburg, 2013).

Regrettably, clinical trials have provided evidence showing that PDGFRβ inhibition with imatinib alone has been ineffective. Several clinical data results indeed correlate low pericyte coverage with enhanced metastasis formation and poor patient prognosis (Cooke et al., 2012). Xian et al. (2006) showed that primary pericyte-deficient PDGF-B^ret/ret^ mice have increased tumor cell metastasis in local lymph nodes and distant organs, suggesting a role of pericytes in reducing metastasis formations (Xian et al., 2006).

The controversial observations described by various groups about the favorable or unfavorable results of pericyte depletion may explain the opposite effects of anti-PDGF drugs on vascular remodeling in tumors with high or low levels of PDGF-BB expression.

Confounding data revealed that PDGF-BB-positive tumors contain a significantly higher density of CD31-positive vessels but a markedly reduced number of NG2-positive pericytes compared with PDGF-BB-negative tumors. Moreover, residual NG2+ pericytes in PDGF-BB tumors are disassociated from the vasculature, as opposed to PDGF-BB-negative tumors, where pericytes are associated with blood vessels. Consequently, the vessel architecture becomes increasingly disorganized and leaky, along with increased levels of PDGF-BB release. Although PDGFR-β blockades prevent neo-vasculature formation in both PDGF-BB-positive and -negative tumors, they significantly inhibit metastasis formation by preventing pericyte loss and vascular permeability in high PDGF-BB-expressing tumors, but the same treatment leads to an increased number of cancer metastases in PDGF-BB-non-expressing tumors, thus suggesting that PDGF-BB levels in tumors might be potentially used as a marker to achieve personalized therapy (Hosaka et al., 2016).

To add complexity to this scenario, targeting the PDGF signaling axis can improve hypoxia-induced EMT of cancer cells and subsequent distant metastasis formation. While primary tumor growth in *in vivo* models is reduced with pericyte ablation, the number of circulating tumor cells and lung metastasis is greatly enhanced after PDGFRβ+ cell depletion (Cooke et al., 2012). However, in tumors that have already undergone EMT, such as vascular mimicry-occurring (VM+) tumors, anti-PDGF signaling treatment could decrease tumor cell aggressiveness. *In vivo* studies show increased pericyte number in VM + tumors compared with VM−tumors since VM + tumor cells are believed to influence vessel maturation by recruiting and interacting with pericytes. This increase in pericyte recruitment could result from the induction of elevated levels of PDGF-B. Targeting the PDGF-B signaling axis therefore induces a therapeutic effect in VM + tumor models (Thijssen et al., 2018).

### 6.3 Different anti-angiogenic drugs as a combined strategy

An effective therapeutic protocol provides combined therapies with synergistic or additive effects that improve the prognosis. A widely explored strategy involves combining anti-angiogenic agents with chemotherapy.

For example, olaratumab is a platelet-derived growth factor receptor alpha (PDGFR-α) blocking antibody that received the FDA approval in 2016 for use in combination with doxorubicin for the treatment of adult patients with advanced soft tissue sarcoma (STS) (Shirley, 2017).

Although anti-angiogenic therapy has shown positive outcomes in various human cancers, its effects in most pre-clinical and clinical studies turned out to be transient, resulting in rapid relapse and tumor regrowth (Bergers and Hanahan, 2008; Darvishi et al., 2019). The inhibition of VEGFR and PDGFβ receptor (PDGFRβ) signaling to simultaneously target both endothelial cells and pericytes, respectively, has been investigated to enhance the efficacy of antiangiogenic tumor therapy and overcome the development of drug resistance in tumors. For example, PDGF/NF-κB/Snail axis-induced downregulation of VEGFR-2 expression in ECs leads to the development of anti-VEGF therapy resistance in GMB. It has been demonstrated that dual VEGFR/PDGFR inhibition reduces tumor-associated ECs and improves the survival of GBM-bearing mice (Liu et al., 2018). A higher cytotoxic effect has been shown in glioblastoma cells with the use of a combined therapy with PDGFR and VEGFR inhibitors or a dual targeting pathway compared to a single treatment (Purcaru et al., 2015). The treatment with low-dose bevacizumab added to PDGF-R inhibition using imatinib in a xenograft mouse model increases vascular normalization without incremental collagen IV deposition that normally affects the favorable outcome of VEGF targeting drugs, leading to reduced vascular leakiness and improved perfusion and access for therapy (Schiffmann et al., 2017).

Several tyrosine kinase inhibitors (TKIs) targeting different spectra of tyrosine kinases have been developed as anti-angiogenic therapies. Sunitinib, a multi-kinase inhibitor, targets VEGFR-1/-2/-3, Flt-3, c-Kit, RET, and PDGFRα/β. It is currently approved for the treatment of gastrointestinal stromal tumors (GISTs) and renal cell carcinoma (RCC) (Yu et al., 2023), and it was the first TKI approved for treating advanced pancreatic neuroendocrine tumors (Majidpoor and Mortezaee, 2021; Liu et al., 2023). Sorafenib, a type II multi-targeted inhibitor (Raf, VEGFR-1/-2/-3, and PDGFR), is approved for the treatment of RCC and hepatocellular carcinoma (HCC). Pazopanib, another multi-kinase inhibitor (VEGFR-1/-2/-3, PDGFRα/β, and c-Kit), is also approved for the treatment of RCC (Majidpoor and Mortezaee, 2021; Liu et al., 2023; Yu et al., 2023). Regorafenib inhibits multiple kinases targeting angiogenic (VEGFR1-3, TIE2), stromal (PDGFR-β, FGFR), and oncogenic receptor tyrosine kinases (KIT, RET, and RAF). It is approved for the treatment of HCC patients who have progressed during or after sorafenib therapy (Heo and Syed, 2018). Anlotinib, a novel multi-targeting tyrosine kinase inhibitor (VEGFR, FGFR, PDGFR, and c-Kit), is approved by the National Medical Products Administration (NMPA) in China for the treatment of locally advanced or metastatic non-small-cell lung cancer (NSCLC) patients who develop tumor progression or recurrence after ≥2 lines of systemic chemotherapy (Syed, 2018).

### 6.4 Immunomodulatory effects of anti-angiogenic drugs and their combination with immunotherapy

In addition to its role in angiogenesis, VEGF also modulates tumor-induced immunosuppression, thus suggesting immunomodulatory properties of anti-VEGF therapy and opening up new approaches that involve a combination of anti-VEGF agents with immunotherapy (Majidpoor and Mortezaee, 2021). VEGF-A modulates several immune cells (dendritic cells, myeloid-derived suppressor cells, and tumor-associated macrophages) in tumor-bearing hosts with the goal of accumulating regulatory T-cells while simultaneously inhibiting T-cell tumor-suppressive functions. Based on these assumptions, several clinical trials have been conducted to evaluate the efficacy of anti-angiogenic agents targeting VEGF-A/VEGFR combined with immunotherapy (Bourhis et al., 2021). For example, the combination of bevacizumab with atezolizumab, an immune checkpoint inhibitor (ICI), has recently been approved in NSCLC, and the benefits of the combination treatment have also been demonstrated in hepatocellular carcinoma (Cheng et al., 2019; Garcia et al., 2020; Cheng et al., 2022). For the treatment of hepatocellular carcinoma, combination therapy using anti-PD-1 antibody camrelizumab plus VEGFR2-targeted TKI rivoceranib, in a randomized, open-label, international phase III trial, improved patient progression-free survival and overall survival compared with sorafenib monotherapy (Qin et al., 2023).

Similar to VEGF, PDGF also has immunomodulatory capabilities. PDGF-AB dimer was shown to inhibit dendritic cell maturation by upregulating the expression of C-type lectin-like receptor 2 (CLEC2) and inducing FoxP3+Tregs polarization (Agrawal et al., 2015). Increasing evidence shows that kinase inhibitors adopted two strategies, i.e., inhibition of PDGFR molecular pathways and modulation of immunologic processes. Treatment with imatinib of fibrosarcoma protuberans carrying the fibrosarcomatous transformation (FS-DFSP) induced the accumulation of activated CD3^+^ T cells and CD163+CD14^+^ myeloid cells expressing the CD209 marker in post-therapy lesions (Tazzari et al., 2017). Immunomodulatory effects of imatinib have also been observed in chronic myeloid leukemia, where the drug elicits antigen-specific T-cell responses, thus protecting patients against relapses. Imatinib also boosts interferon α (IFN-α) secretion in NK cells and reduces regulatory T-cell number in patients affected by gastrointestinal tumors. Furthermore, the results highlight an increase in PD-1 expression on T cells in imatinib-treated human gastrointestinal stromal tumors (GISTs). Moreover, the treatment with imatinib of human GIST cell lines abrogates the upregulation of PD-L1 induced by IFN-γ via STAT1 inhibition (Seifert et al., 2017). These data suggest that imatinib in combination with immunotherapies could improve long-term relapse-free survival and prevent the formation of resistant clones (Zitvogel et al., 2016).

Combination strategies of multi-target anti-angiogenesis inhibitors with immunotherapy have shown promising efficacy. Transient treatment with sunitinib increases the pericyte-wrapping blood vessels, which results in tumor vascular normalization and alleviates hypoxia in tumors. This is followed by decreased Treg cells and increased infiltration of CD8^+^ T cells with inhibited TGF-β1 and IL-10 expression and increased levels of CCL-28, IFN-γ, and IL-12. Since sunitinib malate increases the levels of PD-1 and PD-L1 in the TME of tumor-bearing mice, its combined therapy with anti-PD-1 agents displays a significant reduction in tumors compared with either monotherapy, suggesting that it is reasonable to apply anti-PD-1 therapy after sunitinib malate treatment (Li W. et al., 2020). Sunitinib treatment increases the antitumor immunity response in a phase III trial, where a higher PD-L1 level and a lower p62 level were observed in the tumor region of anti-PD-1-treated responder NSCLC patients compared to non-responder patients. Mechanistically, it was discovered that sunitinib regulates the stability of tumor PD-L1 via p62, which binds to PD-L1 and promotes its degradation through its translocation into autophagic lysosomes. Moreover, sunitinib in combination with the cytotoxic T-lymphocyte-associated protein 4 (CTLA-4) monoclonal antibody shows a synergistic antitumor effect by promoting tumor-infiltrating lymphocyte activity in melanoma and NSCLC mouse models (Li H. et al., 2020). Another multi-kinase inhibitor, anlotinib, in ICI combination therapy, achieved favorable clinical outcomes in LUAD and advanced NSCLC patients (Yu et al., 2023). Sintilimab plus anlotinib therapy in a phase II trial has proven to be efficacious and safe as second-line or later therapy for patients with advanced cervical cancer who received unsuccessful prior chemotherapy (Xu et al., 2022).

Other examples of combination therapies showing improvements in clinical trials are pembrolizumab plus axitinib, which increased OS and PFS in a phase III study of metastatic RCC monotherapy (Tamada et al., 2022), lenvatinib with pembrolizumab, which demonstrated significant progress in PFS, OS, and overall response rate (ORR) in patients with advanced RCC (Choueiri et al., 2023), both compared with sunitinib monotherapy, and avelumab plus axitinib for advanced renal cell carcinoma treatment (Larkin et al., 2023).

In addition to anti-PD1/PDL1 axis therapies, other immunotherapies show benefits in combination with multi-target anti-angiogenesis drugs. For example, MD5-1 is an anti-death receptor 5 (DR5) monoclonal antibody that induces tumor cell death mainly via antigen-presenting cells (APCs) and generates tumor-specific effector T cells. In concordance with improved vasculature normalization and alleviated hypoxia, the combination therapy between MD5-1 and sunitinib enhances the number of activated tumor-infiltrating CD8^+^ T cells, suggesting that targeting blood vessels with anti-angiogenic therapy leads to potential benefits for immunotherapy mediated by CD8^+^ T cells and APCs (Tsukita et al., 2018).

The main ICI plus anti-angiogenic agent combinations are listed in Table 2.

**TABLE 2:** Combination therapy using anti-angiogenic agents with immunotherapy.

Combined therapy	Type of cancer	Study type	Reference
Bevacizumab + atezolizumab	Non-small-cell lung cancer (plus paclitaxel and carboplatin) Hepatocellular carcinoma	Approved Phase III	Cheng et al., 2019 Cheng et al., 2022; Garcia et al., 2020
Rivoceranib + camrelizumab	Hepatocellular carcinoma	Phase III	Qin et al. (2023)
Anlotinib + sintilimab	Metastatic cervical cancer Advanced non-small-cell lung cancer	Phase II Phase 1b	Xu et al. (2022) Yu et al. (2023)
Axitinib + pembrolizumab	Metastatic renal cell carcinoma	Phase III	Tamada et al. (2022)
Lenvatinib + pembrolizumab	Advanced renal cell carcinoma	Phase III	Choueiri et al. (2023)
Avelumab + axitinib	Advanced renal cell carcinoma	Phase III	Larkin et al. (2023)
Sunitinib + MD5-1	Breast cancer model	Preclinical	Tsukita et al. (2018)
Sunitinib + anti-PD-1	Hepatocellular carcinoma model	Preclinical	Li et al. (2020b)
Sunitinib + anti-CTLA-4	Melanoma and Lewis lung carcinoma model	Preclinical	Li et al. (2020a)
Anti-VEGF + ACT-based immunotherapy	Melanoma	Preclinical	Shrimali et al. (2010)

### 6.5 New pericyte-targeted therapeutic approaches

As already mentioned, the altered association between ECs and pericytes in the tumor microenvironment results in a local tissue milieu characterized by high interstitial fluid pressure, acidosis, and hypoxia. Such environmental stressors are believed to promote epigenetic reprogramming in tumor-associated EC and pericytes that exhibit markedly different genomic and proteomic profiles compared to their counterparts in normal tissues, thus suggesting the possibility of immunologically targeting tumor-blood vessel-associated antigens (TBVAs) (Fabian et al., 2017). It has been shown that TBVA-targeted vaccines in murine models promote therapeutic CD8^+^ T cell responses that extend overall survival (Zhao et al., 2012). One example of TBVA that, when targeted, showed promising results is delta-like homolog 1 (DLK1). Overexpression of DLK1 has been found in pericytes within human renal cell carcinoma (RCC) biopsies or in murine models of renal carcinomas (RENCA). Interestingly, tumor growth inhibition and increased rates of CD8 cytotoxic tumor infiltrating lymphocyte (TIL) and vascular normalization have been achieved after therapeutic vaccination against DLK1 in RENCA-bearing mice (Chi Sabins et al., 2013).

Unfortunately, it has also been reported that there is a reciprocal regulation between Dlk1 and its homolog, delta-like homolog 2 (DLK2) gene expressions, resulting in a compensatory increase of DLK2 expression in pericytes after therapeutic vaccination against DLK1 in RENCA-bearing mice. For this reason, Fabian et al. demonstrated a higher effect of combined vaccination against DLK1 and DLK2, with increased antitumor benefits when compared with single-antigen vaccination in both RENCA-bearing mice and B16 melanoma tumor model, leading to vascular normalization and higher recruitment and activation of antigen-specific CD8C TIL (Fabian et al., 2017).

Another strategy that has been used to target DLK1 involves the application of the alpha type-1 polarized DC vaccine (aDC1). This approach demonstrated anti-tumor effects in a syngeneic mouse model of colorectal cancer by improving cytotoxic T lymphocyte activity and affecting the tumor vasculature (McCormick et al., 2023).

In addition to DLK1, RGS5 is an endogenous pericyte antigen that is considered a critical mediator of abnormal vascularization. It has been shown that *Listeria monocytogenes* (Lm)-based vaccine targeting RGS5 exhibits potent anti-tumor effects by recruiting functional type-1-associated T cells and reducing tumor blood vessel rates in syngeneic mouse models of CRC. Since CRC progression and survival are dependent on the functionality of blood vessels that oxygenate tumor tissues and mediate metabolic processes, targeting RGS5 could potentially be a promising therapeutic approach against vascularized tumors like CRC (Anderson et al., 2023).

In the field of cancer immunotherapy, chimeric antigen receptors (CARs) targeting specific vascular cell-expressed antigens have become a new revolutionary support in anticancer treatments. In this context, CAR-NK cells in particular showed more selective anticancer activity with less off-tumor toxicity than CAR-T cells. Pericytes are believed to significantly alter stemness, angiogenesis, vessel perfusion, and BBB permeability and affect immune suppression in the GBM microenvironment (Sattiraju and Mintz, 2019). Since a single-cell analysis study highlighted that CD19 is more expressed in brain pericytes than in lung pericytes (Parker et al., 2020), Kong et al. hypothesized that CD19 CAR-NK therapy could be employed in the treatment of glioblastoma. Specifically, they performed CD19 CAR transduction in induced pluripotent stem cell-derived NK (iNK) cells, and they observed that the treatment of GBM-blood vessel assembloid (GBVA) xenografts with CD19 CAR-iNK cells exhibits greater antitumor efficacy (Kong et al., 2024).

In conclusion, given their role in tumor proliferation, invasion, immune-evasion, and metastasis, pericytes are increasingly establishing themselves as a potential target for several anti-cancer therapies. Anti-angiogenic treatment has become an important anti-tumor strategy, especially from the perspective of vascular normalization, but some limitations, such as transient treatment efficacy, encourage researchers to investigate new therapeutic approaches. Combination therapies or immunologically targeted tumor-blood vessel-associated antigens have shown beneficial effects for the treatment of several types of tumors.

A more in-depth understanding of pericyte functions could lead to more therapeutic opportunities for cancer patients.

## Figures and Tables

**Table:** 

**Ang-1**	Angiopoietin 1
**Ang-2**	Angiopoietin 2
**ATP**	Adenosine triphosphate
**ACT**	Adoptive Cell Transfer
**BBB**	Blood-brain barrier
**BTB**	Blood-tumour barrier
**BDNF**	Brain-derived neurotrophic factor
**CNS**	Central nervous system
**CMA**	Chaperone-mediated autophagy
**CCL-, e.g 5,2**	Chemokine (C-C motif) ligand
**CXCL-, e.g. 1,2,3**	Chemokine (C-X-C motif) ligand
**CSPG4**	Chondroitin sulfate proteoglycan 4
**CTC**	Circulating tumoral cell
**CD-, e.g. 4,8,13,146,45,11b,31**	Cluster of differentiation
**CTLA-4**	Cytotoxic T-lymphocyte-associated protein 4
**ECs**	Endothelial cell
**EPT**	Epithelial to Pericyte Transition
**EMT**	Epithelial-to-mesenchymal transition
**FGF**	Fibroblast -growth factor
**FGFR2**	Fibroblast -growth factor receptor 2
**FAK**	Focal adhesion molecules
**GISTs**	Gastrointestinal stromal tumours
**GDNF**	Glial cell-derived neurotrophic factor
**GFAP**	Glial fibrillary acidic protein
**GBM**	Glioblastoma
**HGF**	Hepatocyte growth factor
**HIF-1α**	Hypoxia inducible factor 1alpha
**ICAM-1**	Intercellular Adhesion Molecule 1
**IFN-α**	Interferon alpha
**IFN-γ**	Interferon gamma
**IL-, e.g. 1b,6,8**	Interleukin
**LLC**	Lewis lung carcinoma
**LPS**	Lipopolysaccharides
**MHC-II**	Major histocompatibility complex class II molecule
**MMP-9**	Matrix metalloproteinase-9
**MSCs**	Mesenchymal stem cells
**MDSCs**	Myeloid-derived suppressor cells
**NPC**	Nasopharyngeal carcinoma
**NGF**	Nerve growth factor
**NG2**	Neural/glial antigen 2
**NO**	Nitric oxide
**NSCLC**	Non-small cell lung cancer
**OS**	Overall survival
**PDAC**	Pancreatic ductal adenocarcinoma
**PFT**	Pericyte-to-fibroblast transition
**PPARγ**	Peroxisome proliferator-activated receptor gamma
**PDGF-BB**	Platelet derived growth factor
**PDGFRβ**	Platelet derived growth factor receptor-β
**PD-L1**	Programmed Cell Death Ligand 1
**PD-1**	Programmed Cell Death Protein 1
**PSF**	Progression-free survival
**RGS5**	Regulator of G-protein signalling 5
**RCC**	Renal cell carcinoma
**αSMA**	α-smooth muscle actin
**SMCs**	Smooth muscle cells
**sGC**	Soluble guanylate cyclase
**SLC**	Solute carrier family
**TGFβ**	Transforming Growth factor beta
**TNFα**	Tumor necrosis factor
**TME**	Tumour microenvironment
**TAM**	Tumour-associated macrophage
**TKI**	Tyrosine kinase inhibitor
**VCAM-1**	Vascular cell adhesion protein 1
**VEGF**	Vascular endothelial growth factor
**VEGFR**	Vascular endothelial growth factor receptor
